# *Anagrus breviphragma* Soyka Short Distance Search Stimuli

**DOI:** 10.1155/2015/727098

**Published:** 2015-10-12

**Authors:** Elisabetta Chiappini, Alessia Berzolla, Annalisa Oppo

**Affiliations:** ^1^Istituto di Entomologia e Patologia Vegetale, Università Cattolica del Sacro Cuore, Via Emilia Parmense 84, 29100 Piacenza, Italy; ^2^Sigmund Freud University, 20143 Milano, Italy

## Abstract

*Anagrus breviphragma* Soyka (Hymenoptera: Mymaridae) successfully parasitises eggs of *Cicadella viridis* (L.) (Homoptera: Cicadellidae), embedded in vegetal tissues, suggesting the idea of possible chemical and physical cues, revealing the eggs presence. In this research, three treatments were considered in order to establish which types of cue are involved: eggs extracted from leaf, used as a control, eggs extracted from leaf and cleaned in water and ethanol, used to evaluate the presence of chemicals soluble in polar solvents, and eggs extracted from leaf and covered with Parafilm (M), used to avoid physical stimuli due to the bump on the leaf surface. The results show that eggs covered with Parafilm present a higher number of parasitised eggs and a lower probing starting time with respect to eggs washed with polar solvents or eggs extracted and untreated, both when the treatments were singly tested or when offered in sequence, independently of the treatment position. These results suggest that the exploited stimuli are not physical due to the bump but chemicals that can spread in the Parafilm, circulating the signal on the whole surface, and that the stimuli that elicit probing and oviposition are not subjected to learning.

## 1. Introduction

*Anagrus breviphragma* Soyka (Hymenoptera: Mymaridae) is a generalist, tiny egg parasitoid that develops in leafhopper and planthopper eggs inserted into vegetable tissues (leaves, stems, twigs, and shoots) of different plants, depending on the season [1] and on the different hosts:* Agalliana ensigera* Oman,* Dalbulus maidis* (DeLong and Wolcott),* Chlorotettix fraterculus* (Berg),* Cicadella viridis* (L.),* Ciminius platensis* (Berg),* Dechacona missionum* (Berg),* Exitianus obscurinervis* (Stål),* Hortensia similis* (Walker), and* Xerophloea viridis* (Fabricius) (Hemiptera: Cicadellidae) and* Conomelus anceps* (Germar),* Delphacodes kuscheli* Fennah,* Dicranotropis hamata* (Boheman),* Muellerianella fairmaire* (Perris), and* Peregrinus maidis* (Ashmead) (Hemiptera: Delphacidae) [2–6].

The majority of these hosts are indicated as harmful to a variety of agricultural (food, ornamental, and medicinal) and forestall crops. Their damage is due to the eggs oviposition wounds and to their nutrition punctures or to the transmission of viruses and phytoplasmas.

*Anagrus breviphragma* development (at 20°C) lasts 21–24 days: 3 for the egg stage, 3 for the motionless first instar larva, 6 for the very active second instar larva, 1 for the prepupal stage, and 6-7 for the pupa. Reproduction is anphigonic or parthenogenetic (arrenotokous). Oviposition can take place in host eggs at a different embryonic development, because the active second instar larva can easily disrupt the embryo tissues [7].

*Anagrus* is one of the most important mymarid genera for biological control, both when it is used in classical biological control programs, introducing laboratory bred parasitoid specimens, and when it keeps pests below damaging levels, in landscape management programs or in natural ecosystems.

*Anagrus breviphragma* is a facultative gregarious parasitoid; in so far as in larger Cicadellidae eggs, five to eight adults per host egg can emerge, while in smaller Delphacid eggs, a single individual per egg develops [7]. This behaviour seems to be determined by the competition between the larvae that hatch from the eggs, often supernumerary within a single host, and not by a decision of the ovipositing female [8]. This superparasitisation has been observed both in the case of the same female and in the case of different females.

*Anagrus breviphragma* is attracted to the infested plant thanks to induced plant VOCs (synomones) and host egg kairomones [9]. Once the female is on the leaf, the typical searching and selection behaviours (“standing still,” “walking while tapering,” “brushing the club,” “drilling,” and “vibrating the abdomen”) have been described [10, 11] even if the specific stimuli that generate them have not been identified.

*Cicadella viridis* eggs are inserted in the attacked plant tissues in bunches of 5–10 or more, causing a reaction of the vegetal tissues surrounding the eggs or a simple swelling, depending on the attacked organ (leaf, stem, or shoot) and species. Therefore, the scar directly made by the leafhopper ovipositor, usually smeared with a transparent substance [1, 7], is widen, revealing the eggs just below, or it is sealed and situated far from the eggs, that is, along the leaf margin. In such a variegated situation, both physical and chemical stimuli could reveal the presence of the host to the searching parasitoid female. Physical cues could be coupled with the swelling due to the eggs' presence or the lump due to the proliferation of the vegetal cells and could be perceived by the parasitoid with mechanical or visual receptors. Other physical stimuli could be due to the scar and to the exposed eggs. In fact,* C. viridis* eggs are equally parasitized whether partially embedded in* Ranunculus acer* L. (Ranunculaceae) stems and* Alnus glutinosa* (L.) (Betulaceae),* Fraxinus excelsior* L.,* Ligustrum vulgare* L. (Oleaceae),* Rosa* spp. (Rosaceae), and shoots [1] or completely hidden in between* Carex riparia* Curtis (Cyperaceae) leaf epidermis [7].

Though the stimuli utilized by ovipositing females in hosts searching behaviour are well known for exposed eggs parasitoids [12–18], both long- and short-range cues are poorly studied for what concerns parasitoids of embedded eggs [12], such as* A. breviphragma*. On the basis of previous, occasional observations, such as the fact that females oviposit also from the flat surface of* Carex* leaves (unpubl. data), and analysing the different oviposition sites exploited by* A. breviphragma*, we hypothesized that physical stimuli are less likely to be used by* A. breviphragma* ovipositing females.

Therefore, in the present study, we investigated the nature of the cues (physical or chemical) that make the female locally search for and recognise the host, probe, and eventually oviposit. In addition, considering the ability of parasitoids to adapt their response to cues based on previous experiences associated with the host presence, particularly significant in generalist parasitoids [19], we examined if the oviposition behaviour can be influenced by the female's learning.

## 2. Methods

### 2.1. Biological Material Origin

All* C. viridis* eggs used in this study were obtained from field-collected material. Leaves of* C. riparia*, cut at their base and bearing overwintering eggs of* C. viridis* in uncultivated areas along the Po river in Piacenza, Italy, were collected periodically during the winter months, from November 2013 to February 2014.

Bundles of about 20–30 leaves were wrapped with wet paper towels, placed in a closed plastic bag, and stored in a refrigerator at 1–3°C. The towelling was changed biweekly to avoid the development of mould.

Parasitised* C. viridis* overwintering eggs were the source of* A. breviphragma*.

### 2.2. Parasitoid Breeding

As overwintering* C. viridis* eggs could be already parasitised by* Oligosita* spp. or* Anagrus* spp. [8] and as, at an early phase, parasitisation is not detectable when the eggs are embedded in the leaf tissues, in our experiments, we used eggs extracted from leaves. In this way, it was possible to check them under a light stereomicroscope to ensure that they were healthy eggs and to confirm that they were all at the same stage of development, namely, embryos without developed eyes.

The extracted eggs, as well as parasitoid adults, were conserved in Petri dishes on wet tissue paper discs, in a conditioned chamber at 20°C, and a long day photoperiod of LD 16 : 8.

Females that emerged from eggs collected in the field were observed under a light stereomicroscope for identification [7]. Those belonging to* A. breviphragma* were put in a Petri dish with conspecific males, white sugar very fine crystals, and healthy* C. viridis* eggs placed on wet tissue paper discs and removed after 24 hours.

Five days later (at second larval instar [20]) parasitised eggs were isolated on wet tissue paper discs in Petri dishes.

After 13/14 days, males and females of the same age emerged and these were used for the tests.

### 2.3. Oviposition Tests

Preliminary video recording of oviposition behaviour of* A. breviphragma* females on host eggs demonstrated that this parasitoid is too small for automatic image analysis. Therefore, as black dots become visible on the egg shell within about 15 minutes from the ovipositor puncture [7], we decided to rely upon this evidence to verify probing.

Considering that both physical and chemical stimuli could reveal the presence of the host to the searching parasitoid female, we considered treatments that verified each stimulus separately. Parafilm was used to eliminate physical stimuli while solvents were used to remove chemicals from eggs surface. At first, nonpolar solvents were preliminary tested but as in this case the washed eggs became grey and degenerated, polar ones were used instead.

The following treatments were then considered:

 Eggs extracted from leaf were used as control since these are frequently utilized for laboratory parasitoid breeding [9] and can mimic those partially exposed (untreated-U).
 Eggs extracted from leaf, cleaned with a synthetic brush in distilled water for 3 minutes, washed in ethanol for 2 minutes, and rinsed again in distilled water were used to evaluate the presence of polar chemicals (washed-W).
 Eggs extracted from leaf and completely covered with Parafilm (M) (Pechiney Plastic Packaging Inc., Chicago, Illinois) in such a way that it was impossible for the parasitoid female to reach the eggs were used to avoid physical stimuli due to the bump on the leaf surface, in correspondence with the eggs, the presence of a scar or a lump, and the possibility to “see” the eggs directly or by transparency (Parafilm-P). As Guerra et al. [21] reported that Parafilm alone induced probing (but not oviposition) in* Catolaccus grandis* (Burks) (Hymenoptera: Pteromalidae), notwithstanding the fact that* Anagrus* is a completely different parasitoid, we previously checked, under a light stereomicroscope for two hours, 10* Anagrus* females on a Parafilm surface without any host egg beneath, but probing was never observed.

Small Petri dishes (⌀ = 1 cm) were prepared twelve hours prior to the experiment starting with wet tissue paper discs and six healthy* C. viridis* eggs just extracted from leaf tissue for each replicate. The Parafilm treatment was prepared cutting a circle of Parafilm just the same size of the Petri dish, placing the wet tissue paper disc on it, positioning the six eggs on the wet paper, covering them with stretched Parafilm and sealing it to the layer underneath. In this way the eggs were closed in a sort of Parafilm pocket with the upper Parafilm surface (on which the wasp was positioned) completely smooth (without any bump). At the beginning of the experiment, one naïve, one-day-old female, left with a male, water, and sugar for the previous 24 hours, was introduced in the Petri dish with eggs.

Two different experiments were performed.

**Experiment 1 (one female was offered one treatment).**

This experiment was performed in order to evaluate the influence of the different stimuli on probing and oviposition behaviours.

Each female was checked every 15 minutes under a light stereomicroscope to detect the eggs with dots in order to obtain the probing start time. After the first dot appeared on the egg chorion, the following checks were performed 5 and 24 hours from the beginning of the test to record the number of eggs with dots per each female. If no dots were observed within 5 hours, the female was left with the eggs until 24 hours from the beginning of the test and checked for dots afterwards. Twenty-four hours after the beginning of the test, the female was removed. In order to confirm parasitisation, we kept the Petri dishes with the eggs on wet tissue paper discs, in a conditioned chamber at 20°C, with a long day photoperiod of LD 16 : 8, until second instar larvae were visible. Fifty replicates were performed per treatment.

**Experiment 2 (one female was offered all of the treatments in sequence).**

This experiment was performed in order to evaluate the influence of a possible learning activity on probing and oviposition behaviours.

Each female was checked every 15 minutes under a light stereomicroscope to record the number of eggs with dots. After two hours on each treatment it was moved to the following one. Six different sequences were possible: U-W-P, U-P-W, W-U-P, W-P-U, P-U-W, and P-W-U. Each of them lasted 6 hours; after this period, the female was removed. In order to confirm parasitisation, the Petri dishes with the eggs were kept in a conditioned chamber at 20°C (photoperiod of LD 16 : 8) until second instar larvae were visible. Ten replicates were performed per sequence.

### 2.4. Statistical Analysis

Data are presented as* N* (%) for categorical data with 95% confidence intervals and as mean (SD) for continuous data (eggs number). Chi-square was used to evaluate the differences between categorical variables using Fisher's exact test where appropriate, while the independent* t*-test and one-way analysis of variance (ANOVA) were applied, where appropriate, to investigate the differences between continuous variables. Paired sample* t*-test and McNemar test were used to analyse differences within groups in continuous and categorical variables, respectively. Cochran's* Q* was used for repeated measures when more than two comparisons were made.

The logistic regression model was used to determine which treatment (between subjects) was associated with a greater percentage of oviposition. In order to check for a possible sequence effect that may cause differences in the number of parasitised eggs in experiment 2, repeated measures ANOVA using one between factor (first treatment presented) in the model was performed assessing possible differences within subject.

Kaplan-Meier survival estimates were used to analyse the hazard of probing in* Anagrus* allocated to the three different treatments: untreated, washed, and Parafilm. The time period considered in the survival analyses was 5 hours in order to analyse the effectiveness of treatment; therefore, data were censored at 5 hours.

All of the post hoc tests were adjusted for multiple comparisons using Bonferroni correction.

The alpha level was set at 0.05. Analyses were carried out using SPSS, version 20.

## 3. Results

Second instar larvae were recognised in all of the eggs with black dots. This result indicates that there was always oviposition after probing, thus confirming that it was correct to rely on black dots to assess probing.

**Experiment 1 (one female was offered one treatment).**

**Figure 1:**
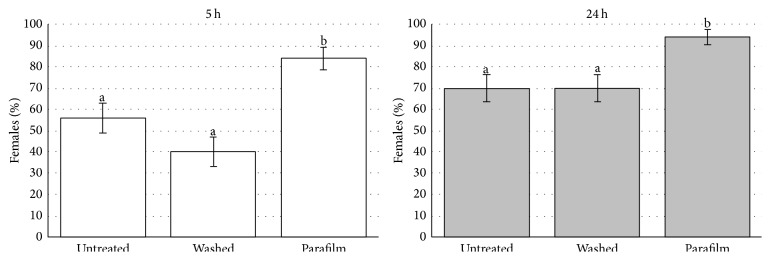
Percentage of females which probed at least one egg in the three considered treatments in 5 or 24 hours' time from the beginning of the test. Vertical lines indicate 95% CIs. In both considered times (5 and 24 h). Bars not sharing the same letter differ significantly (*P* < 0.05).

The percentage distribution was not homogeneous at either 5 hours (chi-square_2_ = 20.66; *P* < 0.001) or 24 hours (chi-square_2_ = 11.19; *P* = 0.004) (Figure 1). At 5 hours, the percentage in “Parafilm” was higher than those in “washed” (chi-square_1_ = 20.54; *P* < 0.001) and in “untreated” (chi-square_1_ = 9.33; *P* = 0.002), while there was no difference between “washed” and “untreated” (chi-square_1_ = 2.56; *P* = 0.11). At 24 hours, post hoc analysis showed similar relationships between treatments. The percentage in “Parafilm” was higher than those in “washed” (chi-square_1_ = 9.76; *P* = 0.002) and in “untreated” (chi-square_1_ = 9.76; *P* = 0.002), while there was no difference between “washed” and “untreated” as the percentage in both was exactly the same (Figure 1).

In all treatments, the percentage of females which probed at least one egg increased from 5 to 24 hours, but this increase was not significant in “Parafilm” (McNemar; *P* = 0.063). However, we had to consider a “ceiling effect” as, in this treatment, the percentage reached almost 100%. On the other hand, there was a significant difference both in “washed” (McNemar; *P* < 0.01) and “untreated” (McNemar; *P* = 0.016).

To determine the relationship between treatments and the likelihood of oviposition, two logistic regression models were used. In the first model, we used oviposition within 5 hours as the outcome measure and found a fourfold higher oviposition likelihood in “Parafilm” compared to “untreated” (OR = 4.13; 95% CI: 1.16–10.55). In the second model, we used oviposition within 24 hours as the outcome measure and found a sixfold higher oviposition likelihood in “Parafilm” compared to “untreated” (OR = 6.71; 95% CI: 1.80–24.99).

The average distribution of parasitised eggs per female in the three considered treatments is not homogeneous at 5 hours (one-way ANOVA; *F*_2,147_ = 31.31; *P* < 0.001) or 24 hours (one-way ANOVA; *F*_2,147_ = 15.62; *P* < 0.001).

**Figure 2:**
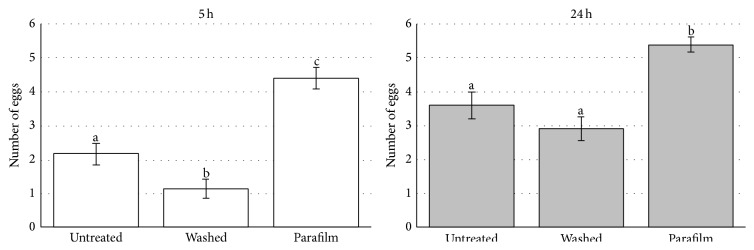
Average number of parasitised eggs per female in the three considered treatments in 5 or 24 hours' time from the beginning of the test. Vertical lines indicate standard deviation. In both considered times (5 and 24 h). Bars not sharing the same letter differ significantly (*P* < 0.05).

At 5 hours, the average number of parasitised eggs per female in “Parafilm” was higher than those in “washed” (*t*_49_ = 3.26; *P* < 0.001) and in “untreated” (*t*_49_ = 3.24; *P* < 0.001), and there was also a significant difference between “washed” and “untreated” (*t*_49_ = 2.47; *P* < 0.015). At 24 hours, the percentage in “Parafilm” was again higher than those in “washed” (*t*_49_ = 2.50; *P* < 0.001) and in “untreated” (*t*_49_ = 1.82; *P* < 0.001), while there was no difference between “washed” and “untreated” (*t*_49_ = 1.30; *P* = 0.196) (Figure 2).

The average number of parasitised eggs per female at 5 and 24 hours significantly increases in all treatments: “Parafilm” (*t*_49_ = 4.25; *P* < 0.001), “washed” (*t*_49_ = 6.10; *P* < 0.001), and “untreated” (*t*_49_ = 5.98; *P* < 0.001).

**Figure 3:**
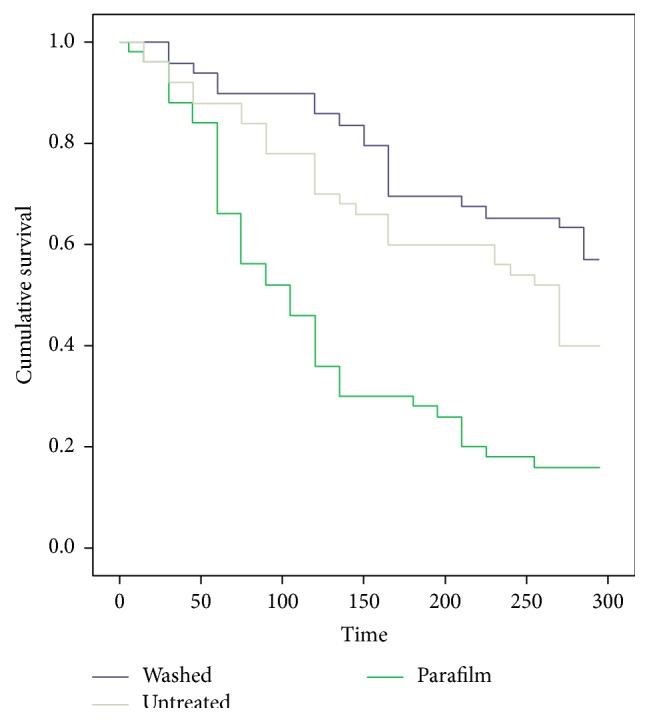
Kaplan-Meier probing starting time (minutes) curves for the three considered treatments at 5 hours.

The probing start time was very variable among females in all of the treatments (in “Parafilm” it varies from 5′ to 300′, in “washed” from 30′ to 300′, and in “untreated” from 15′ to 300′). Nevertheless, the Kaplan-Meier analysis on probing start time in the three considered treatments shows that the “Parafilm” curve was significantly different from the other two (median time = 238′ for “washed,” 208′ for “untreated,” and 130′ for “Parafilm”) (Figure 3).

**Experiment 2 (one female was offered all of the treatments in sequence).**

**Figure 4:**
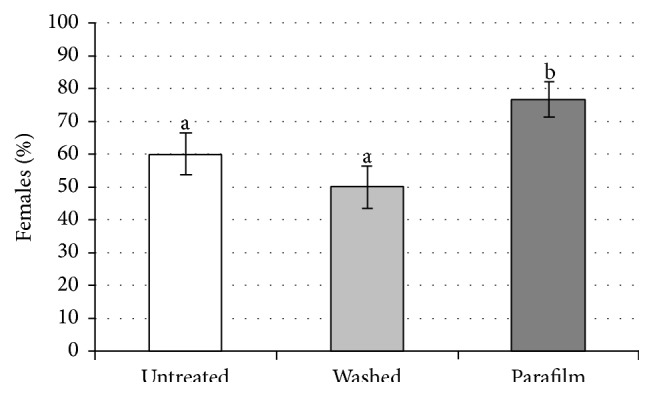
Percentage of females (*n* = 60) which probed at least one egg in the three considered treatments, independently from the sequence in which they had been presented to the females (U-W-P, U-P-W, W-U-P, W-P-U, P-U-W, and P-W-U). Vertical lines indicate 95% CIs. Bars not sharing the same letter differ significantly (*P* < 0.05).

The percentages distribution of females which probed at least one egg was not homogeneous (Cochran's *Q* = 9.88; *P* = 0.005). The value in “Parafilm” was significantly higher than those in “washed” (McNemar; *P* = 0.008) and in “untreated” (McNemar; *P* = 0.041), while there was no difference between “washed” and “untreated” treatments (McNemar; *P* = 0.286) (Figure 4).

**Figure 5:**
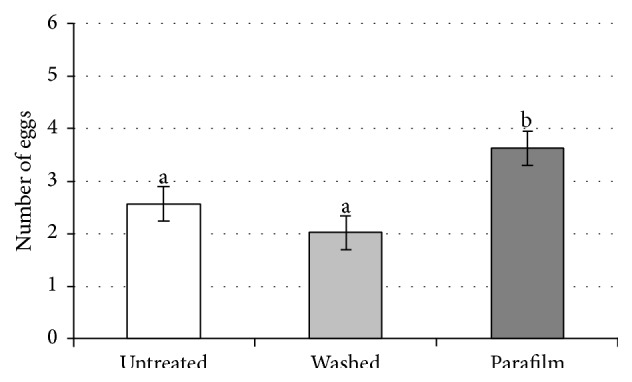
Average number of parasitised eggs per female (*n* = 60 females per treatment) in the three considered treatments, independently from the sequence in which they had been presented to the females (U-W-P + U-P-W + W-U-P + W-P-U + P-U-W + P-W-U). Vertical lines indicate standard deviation. Bars not sharing the same letter differ significantly (*P* < 0.05).

The distribution of the average number of parasitised eggs per female was significantly different (*F*_2,118_ = 7.33; *P* = 0.001) in the three considered treatments. The number of parasitised eggs per female in “Parafilm” was higher than those in “washed” (*t*_59_ = 3.37; *P* = 0.001) and in “untreated” (*t*_59_ = 2.49; *P* = 0.015), while there was no difference between “washed” and “untreated” (*t*_59_ = 1.47; *P* = 0.146) (Figure 5).

The results of the repeated measures analysis of variance show that there was no significant difference due to the first treatment proposed (interaction effect *F*_4,114_ = 0.420; *P* = 0.794).

Indeed, the ovipositing female always preferred the “Parafilm” treatment, independently from the sequence of treatment presentation (*F*_2,118_ = 7.33; *P* = 0.001).

## 4. Discussion

Results show that* A. breviphragma* females oviposit in untreated eggs extracted from leaf tissues as well as in eggs washed with water and ethanol or covered with Parafilm. Nevertheless, “Parafilm” eggs are always preferred to “untreated” or “washed” eggs with respect to probing starting time and average number of parasitised eggs. As the Parafilm flat surface eliminates all physical cues coupled with the swelling present on the attacked plant surface, or the lump due to cell proliferation, or the scar, or the eggs themselves, these results clearly indicate that* A. breviphragma* females short distance search is determined by chemicals present on the eggs surface and somehow able to cross the Parafilm.

Comparing “untreated” with “washed” eggs, no significant difference relatively to both probing starting time and parasitized eggs is present, except for the number of eggs at 5 hours. This result indicates that these chemicals are not eliminated by washing with polar solvents and should therefore be compounds nonpolar or of intermediate polarity [22]. The fact that there is a significant difference with respect to the number of eggs at 5 hours, besides being canceled at 24 hours, can be explained hypothesizing a partial mechanical removal of oviposition stimulating chemicals due to the brush cleaning. This could also justify the reduced, albeit not significant, attractiveness of the washed eggs.

At the same time, the fact that there is a significantly lower probing start time and higher number of eggs parasitised in “Parafilm” treatment indicates that the presence of a “Parafilm” layer covering the eggs enhances parasitisation. Therefore, these chemicals should be able to form weak bonds with the Parafilm and spread in it, circulating the signal on the whole surface. If so, they could behave in a similar way in the waxy epicuticular layer of the leaf surface as hypothesized for* Trichogramma brassicae* Bezdeko (Hymenoptera: Trichogrammatidae) parasitizing* Pieris brassicae* L. (Lepidoptera: Pieridae) eggs on Brussels sprouts plants [23].

Long-range search, which recruits the parasitoid to the attacked plant, providing it with precise information that lets it find the leaf where the host eggs are present, is determined by “a synergistic effect of induced plant VOCs and host egg kairomones” [8]. Once the female has landed on the leaf surface, a short distance search starts with “walking while tapering” and “brushing the club” behaviours [11]. Gustatory sensilla, represented by setae (trichodea) and corresponding to those described for* Anagrus atomus* [24], are disposed on the ventral surface and on the tip of the club of* A. breviphragma* female [9]. These sensilla allow the female to perceive the chemicals which are present on the surface beneath her. Therefore, our results are consistent with the behaviours performed by the female before probing its sensilla type and position. The chemicals that elicit such behaviour could be egg kairomones as those exploited by parasitoids of nonembedded eggs [25] and/or plant synomones locally produced as a reaction to oviposition [19]; in this case, especially, learning should be considered [26].

This hypothesis is confirmed by the fact that the curve of probing starting times is lower in “Parafilm.” In fact, if the chemicals are “absorbed” and spread in this matrix, the probability that a female tapering on the Parafilm surface perceives the chemicals is higher than that in the other treatments.

After probing, the female can decide whether to oviposit or not. It does so when it perceives something (probably host yolk or haemolymph) [8] that is suitable for larval development; in fact, all probed eggs were parasitised.

In experiment 2, both the percentage of females which probed at least one egg and the number of parasitised eggs are significantly higher when the host eggs are covered with Parafilm, even when the naïve female had experienced uncovered eggs first (“untreated” and “washed”). Thus, this behaviour is not subjective to learning, as eggs under Parafilm were always more probed and parasitised, regardless of which treatment was proposed first. These results appear to be in contrast with the idea that generalist parasitoids should get advantage by exploiting conditioned stimuli [26]. Nevertheless, when “Parafilm” is offered first, the females parasitize more eggs in both “untreated” and “washed” treatments, even if not significantly. This could indicate that the females which had experienced the “Parafilm” treatment acquired a useful knowledge in relation to host finding ability. Therefore, after characterizing the chemicals involved, further research should focus on this aspect, also considering whether a specific sequence, not only in relation to the first treatment proposed, may cause an alteration of probing and oviposition behaviour.

## 5. Conclusions

This research allowed us to verify that the short-range cues exploited by* A. breviphragma* females to locate the host are not physical but chemical. Therefore, after characterization of these compounds, a manipulation of* A. breviphragma* oviposition behaviour could be feasible under laboratory conditions. This is very important especially in view of artificial breeding of the parasitoid for research or large number production and commercialization for biological control. In the past, we achieved promising results on* in vitro* rearing of* A. breviphragma* on diets devoid of insect components [20, 24]. The possibility to obtain oviposition through the Parafilm surface treated with chemicals that induce search behaviour and probing should consistently increase the realistic opportunity to rear parasitoids on artificial media.
